# The N-Myc Down Regulated Gene1 (NDRG1) Is a Rab4a Effector Involved in Vesicular Recycling of E-Cadherin

**DOI:** 10.1371/journal.pone.0000844

**Published:** 2007-09-05

**Authors:** Sushant K. Kachhap, Dennis Faith, David Z. Qian, Shabana Shabbeer, Nathan L. Galloway, Roberto Pili, Samuel R. Denmeade, Angelo M. DeMarzo, Michael A. Carducci

**Affiliations:** Prostate Cancer Program, The Sidney Kimmel Comprehensive Cancer Center, Johns Hopkins University School of Medicine, Baltimore, Maryland, United States of America; Max Planck Institute of Molecular Cell Biology and Genetics, Germany

## Abstract

Cell to cell adhesion is mediated by adhesion molecules present on the cell surface. Downregulation of molecules that form the adhesion complex is a characteristic of metastatic cancer cells. Downregulation of the N-myc down regulated gene1 (NDRG1) increases prostate and breast metastasis. The exact function of NDRG1 is not known. Here by using live cell confocal microscopy and *in vitro* reconstitution, we report that NDRG1 is involved in recycling the adhesion molecule E-cadherin thereby stabilizing it. Evidence is provided that NDRG1 recruits on recycling endosomes in the Trans Golgi network by binding to phosphotidylinositol 4-phosphate and interacts with membrane bound Rab4aGTPase. NDRG1 specifically interacts with constitutively active Rab4aQ67L mutant protein and not with GDP-bound Rab4aS22N mutant proving NDRG1 as a novel Rab4a effector. Transferrin recycling experiments reveals NDRG1 colocalizes with transferrin during the recycling phase. NDRG1 alters the kinetics of transferrin recycling in cells. NDRG1 knockdown cells show a delay in recycling transferrin, conversely NDRG1 overexpressing cells reveal an increase in rate of transferrin recycling. This novel finding of NDRG1 as a recycling protein involved with recycling of E-cadherin will aid in understanding NDRG1 role as a metastasis suppressor protein.

## Introduction

Epithelial to mesenchymal transition is central to many physiological and pathological processes including embryogenesis, wound healing and metastasis [1]. Clinically the development of cancer into an aggressive and life threatening disease is determined by its potential and propensity to metastasize [2]. Metastasis involves intravasation from site of origin into the circulatory system and extravasation to the secondary sites. The whole process is brought about by a complex interaction of the tumor cell with its microenvironment [3]. An initial step of metastasis in solid tumors involves downregulation of epithelial cell adhesion molecules, thereby increasing the motility and invasiveness of tumor cells [4].

The adherens junction in epithelial cells is characterized by the E-cadherin complex. E-cadherin is a 120 KD transmembrane glycoprotein present on the surface of epithelial cells [5]. The extracellular domain of the molecule forms a homodimer with E-cadherin of neighboring cells in the presence of extracellular calcium. The cytoplasmic domain of E-cadherin interacts with α, β and γ catenins [6]. It is thought that the complex interacts with the actin cytoskeleton via the α-catenin molecule. However, recent work by Yamada et.al., contests this notion and places the E-cadherin complex as a more dynamic entity enabling morphogenetic changes and tissue development [7]. Loss of E-cadherin by deletion or gene silencing is among the most common adhesion molecule alteration in several cancer types that includes those of the bladder, stomach, breast, colon, kidneys and prostate [8], [9].

The N-myc down regulated gene 1 (NDRG1) has been reported to be a prostate and breast cancer metastasis suppressor gene [10], [11]. NDRG1 gene is conserved in a wide variety of multicellular organisms including *Caenorhabditis*, *Xenopus*, *Drosophila* and sun flower indicating that it may play a role in an essential biological process [12], [13].

The fact that germline mutations of NDRG1 in humans and NDRG1 knockout mouse models do not develop cancers suggests that NDRG1 may not be involved with cancer initiation but at a later event in cancer progression which includes metastasis [14], [15]. However, the exact function by which NDRG1 suppresses metastasis is unknown. It is expressed in a wide variety of tissues including the prostate [16]. Overexpression of NDRG1 leads to differentiation of cancer cells. In experimental settings NDRG1 is induced by a wide variety of chemicals including histone deacetylase inhibitors and differentiating agents like retinoic acid [17]. By immunohistochemical analysis of various human tissues NDRG1 has been shown to localize to nucleus and cytoplasm as well as close to the adherens junctions [16]. It is hypothesized that NDRG1 plays a role in stabilizing the adherens junctions [16]. In this report evidence is provided that NDRG1 is a Rab4a effector protein that localizes to perinuclear recycling/sorting vesicles in the Trans Golgi network by binding to phophatidylinositol 4-phosphate and is involved in recycling of E-cadherin. This is the first demonstration providing evidence that NDRG1 is a Rab4a effector recruiting to recycling/sorting endosomes.

## Results and Discussion

**Figure 1:**
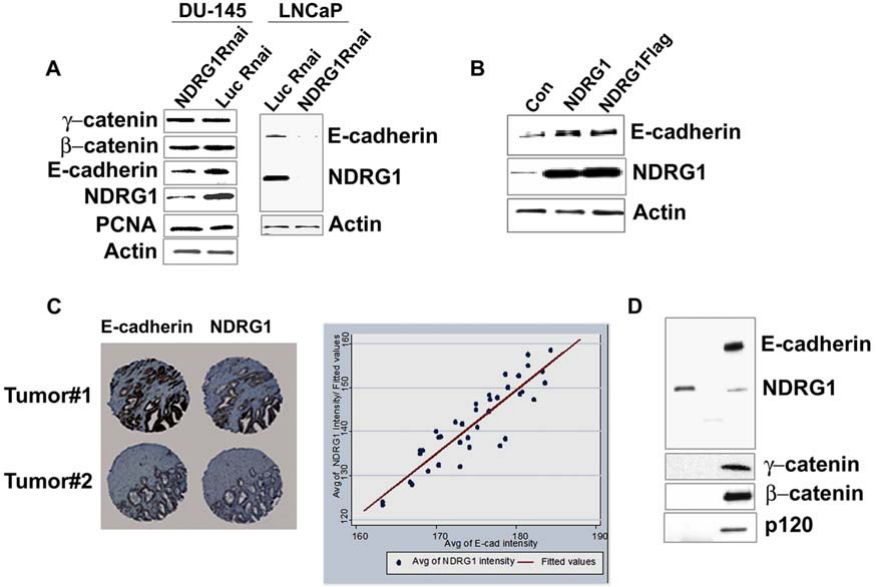
Presence of NDRG1 stabilizes E-cadherin in prostate cancer cells. (A) Western blot analysis of DU-145 and LNCaP cells transfected with pSHAG1NDRG1 constructs. Control cells were transfected with pSHAG1Luc vector. Knockdown of NDRG1 downregulates E-cadherin protein levels but not other proteins of the E-cadherin complex or PCNA, actin was used as a loading control. (B) DU-145 cells transfected with pCMVNDRG1 and pCMVNDRG1flag constructs, 48 h post transfection cells were treated with cycloheximide (10 µM) for 6h and probed for E-cadherin by western blotting. E-cadherin is stabilized in cells overexpressing NDRG1 or NDRG1flag. (C) Immunohistochemical analysis of prostate cancer tissue array shows representative tumors stained for both NDRG1 and E-cadherin. Analysis of average intensity of both the proteins reveal NDRG1 and E-cadherin expression correlates significantly (n = 32, r^2^ = 0.8448). (D) DU145 cells transfected with NDRG1-Flag constructs were lysed with cell lysis buffer, immunoprecipitated and probed for proteins of the E-cadherin complex, none of the probed proteins of the E-cadherin complex interacts with NDRG1. (Figures A, B and D are representatives of at least three independent experiments.)

To investigate a role of NDRG1 in stabilizing the adherence junction, NDRG1 gene expression was knocked down in the prostate cancer cell lines DU-145 and LNCaP using a U6 based promoter shRNA construct. This led to a decrease in E-cadherin protein levels but not other proteins of the E-cadherin complex investigated (Figure 1A). DU-145 cells transfected with NDRG1 or Flag-tagged NDRG1 constructs when treated with cycloheximide, a protein translation inhibitor, showed an increase in E-cadherin levels as compared to mock transfected controls (Figure 1B). This indicates that NDRG1 directly or indirectly stabilizes the E-cadherin protein increasing its longevity in the cell.

To investigate whether there is a correlation between NDRG1 and E-cadherin expression in prostate cancer tissues, both proteins were analyzed immunohistochemically on a prostate cancer tissue array by our published methods [18]. A total of thirty two prostate cancer tumors were evaluated. NDRG1 was found to be primarily cytoplasmic with some membrane and nuclear localization. When individual tumors were analyzed for co-expression of the two proteins, a highly significant positive correlation was revealed (r^2^ = 0.8448, Figure 1C). This finding suggests that presence of NDRG1 may be necessary for the stability of the E-cadherin protein in prostate tumors.

**Figure 2:**
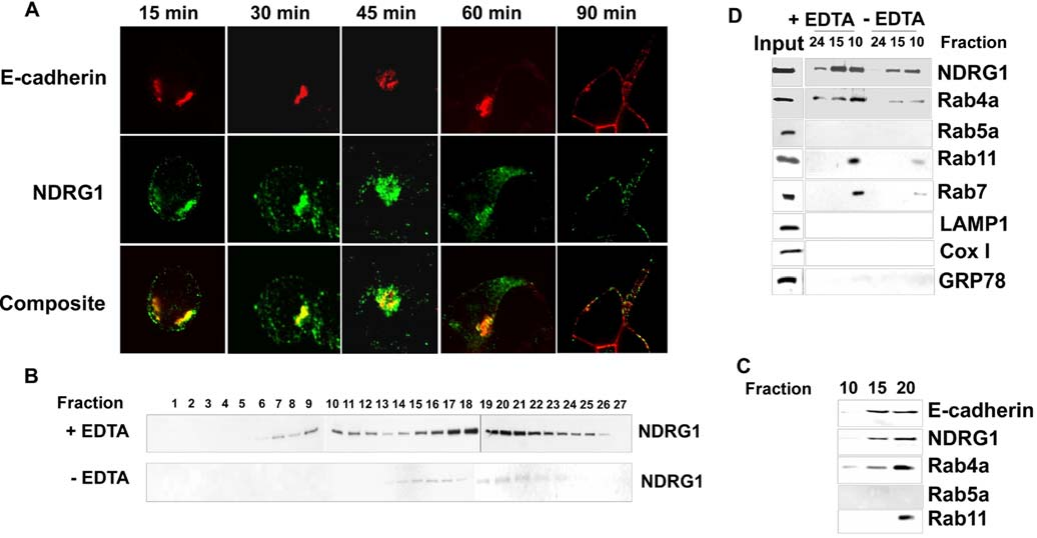
NDRG1 colocalizes with recycling E-cadherin and co-fractionates with recycling endosome. (A) Immunofluorescence analysis of CWR22R cells labeled with E-cadherin antibody and chelated with EDTA. Cells were replated on calcium supplemented media and probed with primary antibody against NDRG1 and secondary antibodies against NDRG1 and E-cadherin after different time intervals. NDRG1 colocalizes with recycling E-cadherin. (B) Organelle fractions (from top to bottom) of DU-145 cells chelated with EDTA and subjected to a sucrose density gradient centrifugation were analyzed by western blotting. NDRG1 strongly localizes to a membrane organelle in cells treated with EDTA. (C) Western blotting of EDTA chelated NDRG1 positive fractions in DU-145 cells reveals NDRG1 cofractionates with E-cadherin (D) Western blotting of NDRG1 positive fractions after sucrose density gradient in HEK293 cells reveals NDRG1 co-fractionates with recycling and late endosomal markers cells after Ca^2+^ chelation. (All figures are representatives of at least three independent experiments.)

Intrigued by such a remarkable correlation between the expression of the two proteins we sought to understand the functional relation, if any, between NDRG1 and E-cadherin. Interaction of E-cadherin with the catenins leads to clustering of E-cadherin thereby strengthening adhesion [6]. To understand the possible role of NDRG1 in stabilizing E-cadherin, we first investigated whether the stabilization was due to association of NDRG1 to the E-cadherin complex. None of the proteins of the E-cadherin complex co-immunoprecipitated with flag-tagged NDRG1 in DU-145 and CWR22R prostate cancer cells, suggesting other mechanisms involving NDRG1 may play a role in the stability of E-cadherin (Figure 2D and data not shown).

E-cadherin has a short half life of 5–10h. The assembly and turnover of the E-cadherin molecule involves its phosphorylation, ubiquitinylation, internalization by endosomes, and subsequent lysosomal or proteasomal degradation or recycle back to the cell surface [19]. This vesicular to and fro movement of E-cadherin from the cell surface to the interior and back is central to the dynamics and stability of the adhesion complex [20], [21]. Given the fact that NDRG1 is induced by calcium ionophores [22] and E-cadherin turnover is calcium dependent, the location of NDRG1 and E-cadherin in DU-145 cells was investigated after calcium chelation and subsequent recovery in calcium supplemented media by immunoflourescence. Our immunofluorescent data revealed that NDRG1 strongly localizes with E-cadherin during the recovery/recycling phase (Supplementary Figure S1). However, due to scanty cytoplasm and large nucleus of DU-145 cells it was hard to discern, even with very thin Z-sections, whether the co-localization seen was apparent or real.

To confirm whether NDRG1 indeed colocalizes with recycling E-cadherin and also test whether this is true in other cells, live CWR22R prostate cancer cells were labeled with mouse monoclonal antibody that recognizes the extracellular domain of E-cadherin. Cells were labeled on ice to prevent endosomal internalization of the antibody and then immediately chelated with EDTA before placing them back to 37°C. Cells were then restored to calcium supplemented media and immunoprobed at different timepoints with antibody against NDRG1 and secondary anti-mouse antibody to detect endocytosed recycling E-cadherin. Punctate staining for NDRG1 was observed in the cytoplasm. Intense staining for both the proteins was seen near the perinuclear region where the two proteins colocalized. As the cells spread, structures positive for both the proteins resolved into more tubular-vesicular morphology and NDRG1 was seen to colocalize with recycling E-cadherin (Figure 2A).

Vesicular transport in the cell is guided by small ras-like GTPases or RabGTPases of ∼20–30 KD that shuttle between the cytoplasm and vesicle membranes. Together the different Rab proteins act as molecular switches that spatially and temporally regulate protein trafficking, recycling, and degradation [23]. Endocytosed surface molecules and receptors pass through vesicles decorated by different Rab proteins and effectors before they are degraded or recycled back to the cell surface [24]. Rab11 is known to be associated with recycling E-cadherin [25].

Does NDRG1 play a role in vesicular transport was the next question addressed. A discontinuous sucrose density ultracentrifugation was employed to reveal the localization of NDRG1 in DU-145 cells after calcium chelation using EDTA. Fractions collected and subjected to western blotting revealed that NDRG1 strongly localizes to a membrane organelle in the presence of EDTA (Figure 2B and 2C). These fractions were positive for E-cadherin (Figure 2C). However, the presence of E-cadherin was evident only when an increased amount of each fraction was loaded indicating all organelles positive for NDRG1 were not positive for E-cadherin. That this is a common feature of all cells and not specific for a particular cell type was confirmed when the same findings held true in adenovirus transformed human embryonic kidney (HEK293) cells (Figure 2D).

**Figure 3:**
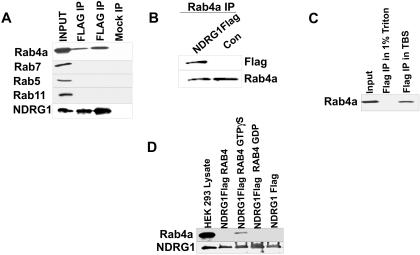
NDRG1 interacts with GTP-bound Rab4GTPase. (A) HEK293 cells transfected with NDRG1Flag vector lysed in Tris-buffered saline, immunoprecipitated using M2-agarose and probed for different RabGTPase by western blotting. NDRG1 interacts specifically with Rab4a. Lanes 2 and 3 are immunoprecipitation from two different experiments. (B) Reciprocal immunoprecipitation using Rab4a antibody and probed with flag antibody detects a single band in NDRG1 flag transfected cell lysates. (C) Immunoprecitation of NDRG1 carried out in cell lysis buffer containing 1% TritonX100 and in Tris-buffered saline (TBS). Binding of NDRG1 to Rab4a is sensitive to TritonX100. (D) NDRG1flag purified from Drosophila S2 cells and bound to M2 agarose were incubated with purified Rab4aGST loaded with GTPγS and GDP. Bound proteins were analyzed by western blotting, NDRG1 specifically binds to Rab4aGST loaded with GTP.

To decipher the identity of the organelle to which NDRG1 localized NDRG1 positive fractions were probed with different organelle markers. NDRG1 positive fractions were positive for, Rab4 and Rab11, (markers for recycling/sorting endosomes) and Rab7 (late endosomal marker) while negative for Rab5a (early endosomal marker), LAMP1 (lysosomal marker), GRP78 (endoplasmic reticulum marker), and CoxI (mitochondrial marker), (Figure 2C and D). This indicated that NDRG1 containing organelles co-fractionates with recycling/sorting and late endosomes. Also, the distribution of NDRG1 and Rab4a was remarkably similar. In order to investigate whether NDRG1 physically associates with Rab4a or other RabGTPases, HEK293 cells were transfected with NDRG1 Flag constructs. NDRG1 specifically immunoprecipitated with Rab4a and this interaction was sensitive to TritonX100 (Figure 3A and 3C). A reciprocal immunoprecipitation using Rab4a antibody under similar condition when performed and probed for flag-tagged NDRG1 revealed a positive interaction between the two proteins confirming our finding (Figure 3B). None of the other RabGTPases (Rab5, Rab11 and Rab7) which are located on distinct endosomal membranes immunoprecipitated with NDRG1 (Figure 3A).

We hypothesized that the interaction between NDRG1 and Rab4a may occur on the surface of endosomal membranes rich in disordered lipids that are sensitive to TritonX100 solubilization [26]. To ascertain this, recombinant flag-tagged NDRG1 protein were generated in S2 *Drosophila* insect cells and purified using a combination of anion-exchange and affinity chromatography. Rab4a was produced as a GST tagged protein in BL21 E.coli cells (Figure S2A and B). RabGTPases shuttle between cytoplasm and endosomal membranes, they are converted to the GTP-bound form by a GTP/GDP- exchange factor and localizes to the membranes of endosomes recruiting Rab effector proteins on the surface of the vesicle. Therefore, recombinant Rab4a was loaded with GTPγS and GDP and incubated with M2-agarose bound purified NDRG1flag protein. NDRG1 specifically interacted with GTPγS-bound Rab4a and not GDP-bound Rab4a indicating that NDRG1 indeed has an affinity for membrane-localized Rab4a (Figure 3D). This suggested that NDRG1 is a candidate Rab4a effector protein. To investigate whether this is true *in vivo* we employed constitutive active and inactive mutants of flag tagged Rab4a. The Rab4aQ67L is GTPase deficient and is constitutively active whereas the Rab4aS22N is GDP-bound and inactive [27].

**Figure 4:**
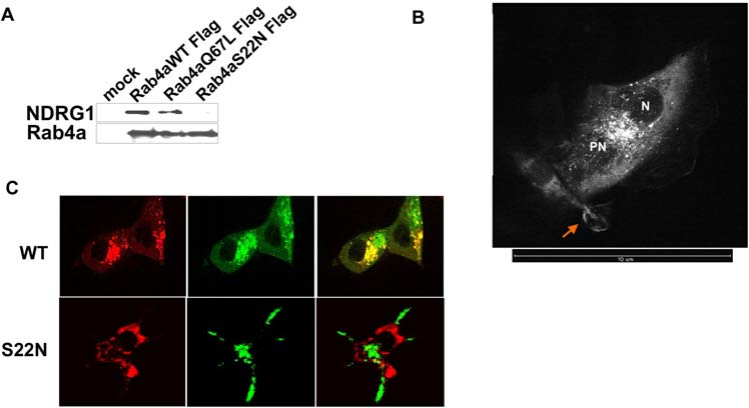
NDRG1 interacts with wild type and Q67L mutant of Rab4a but not with S22N mutant. (A) Immunoprecipitation of flag-tagged wild type and mutant Rab4a reveals NDRG1 interacts specifically with wild type and GTP-bound Q67L mutant of Rab4a. (B) Confocal microscopy of NDRG1DsRed2-HEK293 stable cells reveal NDRG1 localize asymmetrically around the nucleus (N) to discrete perinuclear (PN) vesicles. NDRG1 is also seen to localize to membrane ruffles (arrow). Scale bar indicates 10µm length. (C) Wild type and GDP-bound (S22N) EGFP fusion of Rab4a transfected in NDRG1DsRed2-HEK293 stable cells reveal vesicular NDRG1 colocalizes with wild type Rab4a but not with the S22N mutant.

**Figure 5:**
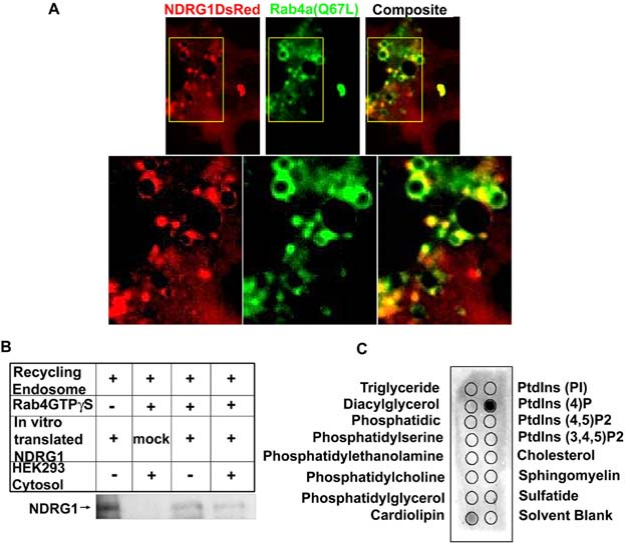
NDRG1 recruits to recycling endosome by binding to phosphatidylinositol 4-phosphate and interacts with Rab4GTPase. (A) NDRG1DsRed2-HEK293 stable cells transfected with Rab4aQ67LEGFP mutant reveal NDRG1 interacts with Rab4aQ67LEGFP at the perinuclear region. (B) *In vitro* translated NDRG1 was incubated with purified recycling endosomes in the presence of recombinant GTPγS bound Rab4a and HEK293 cytosol.NDRG1 recruits on recycling endosomes independent of Rab4a or other cytosolic proteins. (C) Lipid overlay assay using purified NDRG1flag protein (1ug) shows NDRG1 binds strongly to phosphatidylinositol 4-phosphate. (All figures are representatives of at least three independent experiments.)

Immunoprecipitation using these mutants revealed NDRG1 specifically binds to wild type and GTP-bound Rab4aQ67L but does not bind to the GDP-bound Rab4aS22N mutant corroborating our *in vitro* pull-down experiments (Figure 4A). We further validated these findings by transfecting EGFP fusion constructs of wild type and Rab4a mutants in HEK293 cells stably transfected with NDRG1DsRed2 fusion constructs. Stable expression of NDRG1 as a DsRed2 fusion protein showed NDRG1DsRed2 to be primarily a cytoplasmic protein recruiting to discrete vesicles ranging from 30–300nm in size with an average size of 50nm that had an asymmetric distribution in the perinuclear region (Figure 4B, Movie S1). NDRG1DsRed2 also localized to membrane ruffles, a structure whose maintenance is closely associated with endosome function (Figure 4B) [28]. Vesicular wild type Rab4aEGFP was seen to colocalize with NDRG1DsRed2 at the perinuclear region while dominant negative inactive S22N mutant EGFP protein did not colocalize with NDRG1 protein (Figure 4C). The constitutively active Q67L mutant EGFP protein on the other hand showed marked colocalization with NDRG1DsRed2 at the perinuclear region (Figure 5A). For a protein to qualify as a RabGTPase effector it should recognize and interact with GTP bound RabGTPase [23]. Our *in vitro* and *in vivo* data indicates that NDRG1 does qualify as a Rab4a effector protein.

Rab4a is known to recruit many effector molecules on the surface of endosomes [23]. Our pull-down experiment suggested the likelihood of Rab4a recruiting NDRG1 onto the vesicles. However the fact that the dominant negative S22N mutant failed to solubilize vesicular NDRG1 suggested NDRG1 recruitment onto endosomal membranes might be independent of Rab4a protein. To understand the recruitment of NDRG1 on recycling/sorting vesicles, recycling/sorting vesicles were purified by immunoisolation using Rab11 antibody bound to ProteinA magnetic beads from a population of early/recycling endosomes, purified using flotation gradient ultracentrifugation. ^35^S-labeled *in-vitro* translated NDRG1 was incubated with purified recycling endosomes in the presence of GTPγS-bound Rab4a. HEK293 cytosol was included to investigate the role of other cytosolic proteins in the recruitment. NDRG1 recruited onto the surface of endosomes independent of Rab4a or other cytosolic proteins (Figure 5B). This suggests that NDRG1 recruits to recycling vesicles and interacts directly with Rab4a after recruitment.

It is also possible that NDRG1 recruits on vesicles after binding to endogenous membrane-bound Rab4a and does not need any exogenous GTPγS-bound Rab4a protein for membrane recruitment. We speculated that recruitment of NDRG1 onto endosomal membranes may occur by its interaction with lipids on endosomal membranes. Rab5 has been known to recruit phosphatidylinositol kinases that modify lipids and create membrane domains for recruitment of effector proteins [29]. To investigate this possibility purified NDRG1Flag protein from insect cells was used in a lipid-protein overlay analysis. NDRG1 strongly interacted with phosphatidylinositol 4-phosphate (Figure 5C), a lipid concentrated and maintained by ARF1 in the Trans Golgi Network, and is involved with vesicle formation, transportation and sorting cargo proteins to endosomes [30]. However, NDRG1 did bind weakly to cardiolipin, a lipid found in the inner membrane of the mitochondria and its oxidation is involved in induction of apoptosis [31].The biological consequence of NDRG1 binding to cardiolipin remains unknown. Thus, this data indicates that NDRG1 recruits onto vesicles by binding to phosphatidylinositol 4-phosphate and interacts with membrane bound Rab4a.

Further, localization of NDRG1 in live NDRG1DsRed2-HEK293 cells was studied by live cell confocal microscopy. NDRG1DsRed containing vesicles was seen to be motile undergoing both fission and homotypic fusion (Movie S2). NDRG1 vesicles also assume long tubular shapes (5–20 µm in length) and rapidly move from the perinuclear space to the peripheral region near the plasma membrane, a feature that is characteristic of perinuclear recycling/sorting compartment (Movie S2) [32], [33].

**Figure 6:**
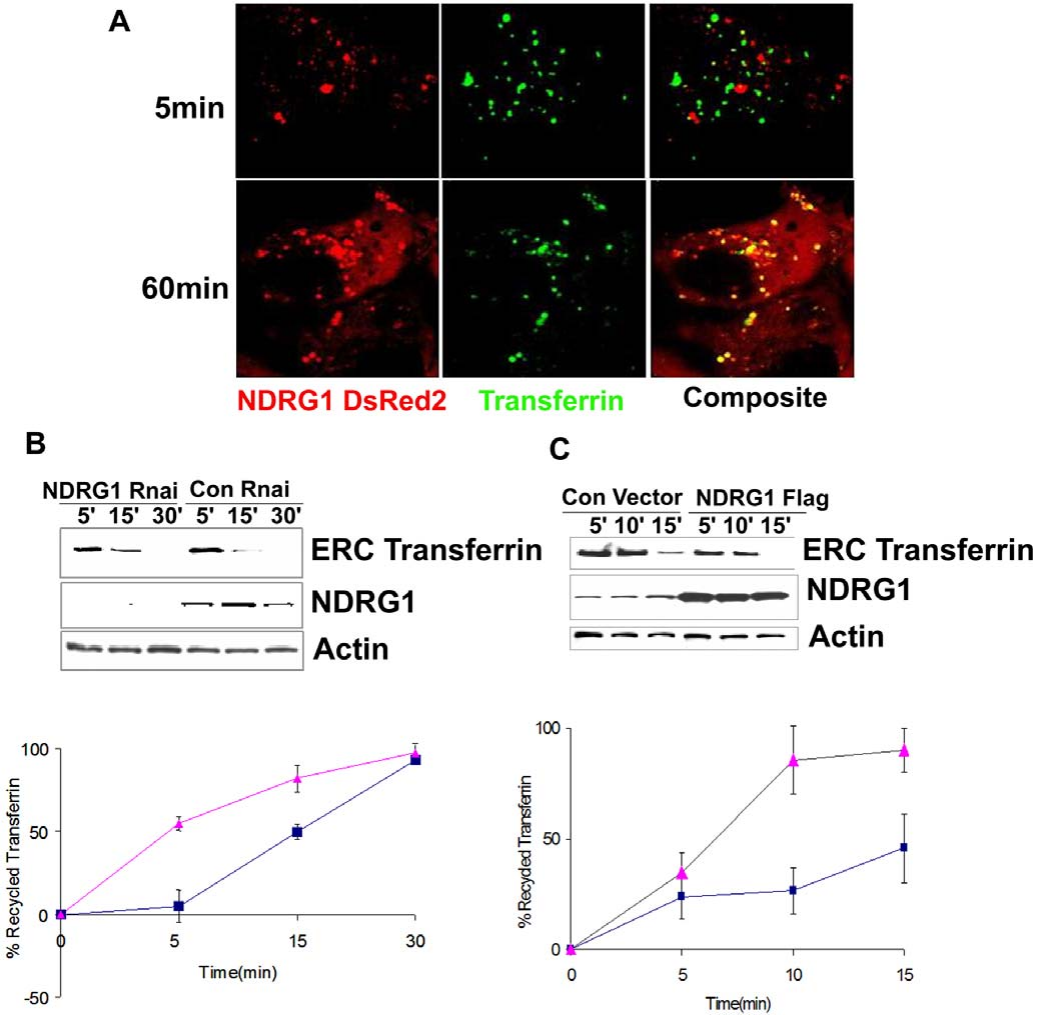
NDRG1 affects transferrin recycling kinetics. (A) Live Cell confocal images of stable NDRG1DsRed2-HEK293 cells pulsed with transferrin for 5 min to load the early endosome and 60 min to load recycling/sorting vesicles reveal NDRG1DsRed2 colocalizes with recycling transferrin. The lower image panel is a still from Movie S3. (B) Transferrin recycling assay in HEK293 cells transfected with NDRG1 shRNA and control shRNA reveal a slower clearance of biotinylated transferrin from the endosomal recycling compartment (ERC) in knockdown cells. The graph below shows the percentage of recycled transferrin (see methods) in the NDRG1 knockdown (▴) as compared to control (▪) cells. The error bars represent standard deviation of 3 independent experiments. (C) Transferrin recycling assay in HEK293 cells transfected with NDRG1flag constructs and empty vector constructs reveal a faster clearance of biotinylated transferrin from the ERC in NDRG1 overexpressing cells as compared to empty control cells. The graph below shows the percentage of recycled transferrin in the NDRG1 overexpressing cells (▴) as compared to control (▪) cells.

To confirm the involvement of NDRG1 in recycling, NDRG1DsRed2-HEK293 cells were pulsed with Alexa-fluor-488 conjugated transferrin for 5min to load the early endosome and 60min to load the recycling endosomes and followed by live cell confocal microscopy. Vesicular NDRG1DsRed2 specifically interacted with recycling transferrin and there was a spatial difference between the transferrin positive early endosomal vesicles that were localized near the plasma membrane and NDRG1 containing vesicles that were localized in the perinuclear region (Figure 6A, Movie S3). NDRG1 vesicles positive for transferrin were seen to recycle transferrin back to the cell surface. To understand the role of NDRG1 in the recycling process we employed transferrin recycling assays on NDRG1 knockdown and NDRG1 overexpressing HEK293 cells. Serum starved cells were loaded with biotinylated transferrin for 1h to load the endosomal recycling compartment and recycling was initiated with excess of transferrin (1 mg/ml). Biotinylated transferrin within the endosomal recycling compartment had a slower recycling rate in NDRG1 knockdown cells as compared to control shRNA vector transfected cells (Figure 6B). This data was also confirmed when recycled transferrin was compared between NDRG1 knockdown and control transfected cells (Figure 6B graph). However, a difference in recycling rates was evident only at early time points (5min and 15min) and NDRG1 knockdown cells were able to recycle most of the endocytosed transferrin after 30 min. Overexpression of NDRG1 in HEK293 cells increased the rate of transferrin clearance from the endosomal recycling compartment as compared to vector transfected control cells (Figure 6C). This was also demonstrated by an increased rate of recycled transferrin in NDRG1 overexpressing cells as compared to vector transfected control cells (Figure 6C graph). Thus both our knockdown and overexpression data demonstrates a functional role of NDRG1 in the recycling pathway. A delay in transferrin recycling has been noted after knockdown or knockouts of a number of protein involved with vesicular transport, specifically proteins belonging to the EHD family that localizes to tubular vesicular regions of recycling endosomes [34]. Interestingly, during the revision of this manuscript a report by Taketomi et al., demonstrated impaired exocytosis and maturation of mast cells in NDRG1 knockout mice. Mast cells from NDRG1 knockout mice displayed 50% less exocytosis and exhibited fewer, smaller and irregular secretory granules as compared to wild type controls [15]. Forced expression of NDRG1 in mast cells by the same group had demonstrated an increase in exocytosis and degranulation [35]. Arguing that the exocytosis process and the endosome recycling pathway shares common protein components and are functionally related, our data demonstrating a delayed kinetics of transferrin recycling in NDRG1 knockdown cells and increase rate of transferrin recycling in NDRG1 overexpressing cells is consistent with the findings of Taketomi et al., [15], [36].

As indicated above NDRG1 containing vesicles ranged in size from 30–300 nm. Large vesicle size (300 nm) suggests that besides recycling, NDRG1 may also have secretory function [37]. After extensive literature search and surveying proteomic data of prostasome, a vesicular body secreted by the prostate that helps in sperm motility, NDRG1 was found to be one of the protein components of prostasome besides other proteins involved in vesicular transport [38]. Recently, by confocal microscopy and BRET analysis, NDRG1 was shown to colocalize with APO AI and AII and may be involved with secretion or transport of these lipoproteins [39]. Interestingly, a plant homolog of NDRG1 is also expressed in secretory cells of reproductive tissues [13]. Although this report does not investigate the role of NDRG1 in secretory pathways, it may not be surprising if NDRG1 also has secretory functions as many proteins of the endocytic and exocytic pathways overlap [15], [36].

**Figure 7:**
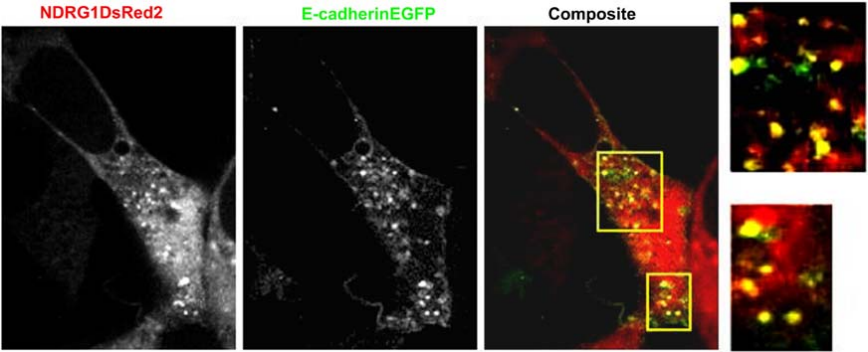
Live cell confocal microscopy confirms NDRG1 involvement with recycling E-cadherin. Live cell confocal images of stable NDRG1DsRed2-HEK293 cells transfected transiently with E-cadherinEGFP construct and plated in calcium-supplemented media after being chelated with EDTA shows NDRG1DsRed2 positive vesicles interact with recycling E-cadherinEGFP both near the perinuclear space and close to the membrane. The image is a still from Movie S4.

Having established that NDRG1 is involved with recycling/sorting endosomes, involvement of NDRG1 with E-cadherin recycling was studied using live cell confocal microscopy. For this purpose an E-cadherinEGFP construct that is known to be functional and which also interacts with cytoplasmic catenins was employed [7]. Transient transfection of E-cadherinEGFP construct in NDRG1DsRed2-HEK293 cells was followed by calcium chelation and recovery in calcium-supplemented media. Live cell confocal images show endocytosed E-cadherinEGFP to be vesicular and are enriched at the perinuclear space before being recycled back to the cell surface. NDRG1DsRed2 vesicles both near the perinuclear space and close to the membrane fuse with E-cadherinEGFP vesicles as they are trafficked back to the cell surface confirming its involvement with recycling E-cadherin (Figure 7 and Movie S4).

Although NDRG1 has been reported to be downregulated in a variety of cancers which includes the cancers of prostate, breast, colon and oesophagus there are also reports that NDRG1 is upregulated in hepatic, pancreatic and kidney cancers [10], [11], [40]–[43]. Induction of NDRG1 in these tumors is speculated to be in response to tumor stress or hypoxia and NDRG1 is proposed as a marker of tumor hypoxia [44]. However, in pancreatic cancer, cellular differentiation and not hypoxia was demonstrated to be the determining factor for NDRG1 expression [42]. In renal cancer, induction of NDRG1 in the tumor tissue was restricted to infiltrating macrophages and not cancer cells [43]. Also none of the reports demonstrate experimental overexpression of NDRG1 leading to increased proliferation or invasion in cellular or animal models of these cancers. Mutation status of NDRG1 in tumors overexpressing NDRG1 is lacking, it is also likely that NDRG1 in these tumors may be non functional.

Apart from an alpha-beta hydrolase motif, the functionality of which remains questionable [45], NDRG1 has no known protein motifs that would impart it a function. Germline mutations in NDRG1 causes Charcot-Marie Tooth Disease type 4D, a demyelinating disorder [14]. Myelin biogenesis involves coordinated activities of both the endocytic and the exocytic pathways [46]. NDRG1 mutation may lead to perturbation in one or both the pathways leading to the observed phenotype. In summary, this report provides evidence defining NDRG1 function in vesicular transport. The data presented here indicates that NDRG1 is a Rab4a effector that can localize to the recycling/sorting endosomes. Being a Rab4a effector it is very likely that NDRG1 may also be involved with vesicular transport of other cargo molecules that may account for its metastasis suppressor function. Our report suggests the involvement of NDRG1 in recycling of at least one metastasis suppressor, the E-cadherin molecule. Also, the fact that NDRG1 and E-cadherin protein levels correlate significantly in tumors from prostate cancer patients renders clinical significance to our *in vitro* findings.

## Materials and Methods

### Constructs

A U6 promoter based vector (pSHAG vector and pSHAGLuc control vector, a kind gift from Dr. Greg. Hannon, Cold Spring Habor) was used to generate shRNA against NDRG1. Three regions targeting the coding region of NDRG1 were designed according to the software (http://katahdin.cshl.org:9331/RNAi) for bases 144–172, 304–331 and 464–491 (NM_006096). For cloning NDRG1 full length gene in a mammalian expression vector NDRG1 was amplified from a prostate cDNA library (Clontech) using reverse primers that had a Flag sequence (DYKDDDDK) in-frame with the NDRG1 coding sequence,a stop codon was introduced after the FLAG sequence. The PCR product was cloned into the XhoI/EcoRI site of the pDsRed2N1 (Clontech) vector and sequenced. The NDRG1 DsRed2 fusion vector was made by amplifying NDRG1 from the prostate cDNA library and cloning it in-frame with the DsRed2 protein. Rab4a wild type and mutant EGFP fusion vectors were a kind gift from Dr. Marci Scidmore (Cornell University). These vectors were used as a template to PCR amplify and generate N-terminal Flag tagged Rab4a and mutant constructs.

### Knockdown of NDRG1

shRNA constructs were transfected in prostate cancer cell lines using Lipofectamine2000 Reagent (Invitrogen) and proteins were harvested 24, 48 and 72 h post-transfection for western blotting. Control cells were transfected with pSHAG1luc constructs that targets the luciferase gene. NDRG1 shRNA construct targeting region 304–331 bases was selected as it gave a maximum knockdown.

### Immunofluorescence and Immunoprecipitation

DU-145 cells were chelated with 2.5 mM EDTA and plated on coverslips in calcium-supplemented media. Cells were fixed at different time points with 3.6% formaldehyde and permeablized with 0.125% TritionX100 before being probed with antibodies against NDRG1 (a kind gift from Dr. Therese Commes, Universite Montpellier II) and E-cadherin (Calbiochem). For live cell labeling and recycling experiment CWR22R cells were grown on 60mm dishes and washed with PBS before being chilled on ice. Cells were labeled with E-cadherin antibody (mouse monoclonal 1ug/ml, Calbiochem) in PBS supplemented with 1% BSA for 30 min. Cells were washed with ice cold PBS for 5 times and restored back to 37°C and 5%CO_2_ environment after addition of RPMI and subjected immediately to calcium chelation by addition of EDTA (2.5 mM). Control cells were subjected similarly without the addition of E-cadherin antibody during labeling. After chelation cells were plated on glass coverslips and fixed at different time points with 3.6% formaldehyde for 1 min at 37°C followed by ice cold methanol for 1min at −20°C. Control and labeled cells were permeabilized with 0.125%Triton X-100 or 0.001% digitonin for 3 min and blocked with 1% BSA in PBS. Cells were probed with NDRG1 primary antibody followed by fluorescent labeled secondary antibodies for the two proteins and imaged. Imaged stacks were processed by the Image J or Volocity software before being made into Tiff/Jpeg files. For immunoprecipitation, flag-tagged NDRG1 and Rab4a contructs were used. Constructs were transfected in cell lines and 72 hour post transfection cells were lysed by passing through a 21 gauge needle in Tris-buffered saline containing protease (Roche) and phosphatase (Sigma) inhibitors. Lysates were cleared by centrifugation and used for immunoprecipitation with M2-conjugated agarose (Sigma). For immunoprecipitations involving E-cadherin complex, lysates were made in cell lysis buffer (20 mM Tris-HCl (pH 7.5), 150 mM NaCl, 1 mM EDTA, 1 mM EGTA, 1% Triton, 2.5 mM sodium pyrophosphate, 1 mM sodium beta glycerophosphate) containing protease and phosphatase inhibitors and then immunoprecipitated using M2-agarose.

### Tissue Array

A prostate cancer tissue array was performed according to our published methods [18]. There were 68 cores of tumor from 32 patients that could be evaluated. Staining for the proteins were carried out using Envision+kit (DakoCorp., Carpentaria, CA). Tissue were also probed for CK8 for automated cellular analysis and counterstained with Gill's hematoxylin in a 1:4.5 dilution. Stained slides were scanned using the ChromaVision ACIS II system (ChromaVision Medical Systems, San Juan Capistrano, CA) and values were normalized with CK8 staining. Spots with a diagnosis of cancer or normal were averaged for each patient in terms of average intensity of membrane staining for E-cadherin and average intensity of overall staining for NDRG1. Briefly, brown thresholds were adjusted to correlate with the immunopositive signal, whereas blue thresholds were adjusted for the nuclear counterstain. Images were excluded from analysis if cores were folded, absent, incomplete, or contained mixed tissue types. A score for stained protein (NDRG1 or E-cadherin) was calculated by dividing the brown area for the protein (in pixels) by the brown area for keratin 8 (stained for epithelial cells) and then multiplying by 100. Data was exported to Stata 8.0 for statistical analysis and analyzed by Wilcoxon rank-sum test and regression analysis.

### Recombinant proteins

Flag tagged NDRG1 was PCR amplified and cloned in pMT/BiP/V5-His vector (Invitrogen). Stable Drosophila S2 transfectants expressing the recombinant proteins were induced with copper sulphate (500 µM) in a serum free media for 4 days. Secreted protein was dialyzed in 10 mM Tris-HCl (pH 7.4) and passed through Q-Sepharose column. Bound proteins were subjected to a step-gradient of NaCl. Eluted protein was adjusted to 150mM NaCl before being affinity purified with M2 agarose (Sigma). Rab4a was amplified from a prostate cDNA library and cloned in PGEX2T vector and produced as a GST fusion protein in BL21 (DE3) cells. Recombinant proteins were confirmed by Western Blotting.

### Pull-Down assay

One microgram of purified Rab4aGTPase was loaded with either 100 mM GDP or 10 µM GTPγS in a HEPES binding buffer (20 mM HEPES, 1 mM MgCl_2_, 1mM DTT and 100 mM potassium acetate) for 30 min at 37° C and incubated with M2 agarose bound NDRG1Flag for 1h. Bound proteins were analysed by Western blotting.

### Transferrin Recycling assay

HEK293 cells were plated in a 12 well plate and transfected with NDRG1shRNA and NDRG1 flag contructs with corresponding control vectors. Forty eight hour post transfection cells were subjected to a transferrin recycling assay [47]. Briefly, cells were serum starved for 1h before the assay by replacing the serum (10%FBS) containing MEM medium with MEM medium supplemented with 2 mg/ml of BSA. After starvation the medium was replaced with BSA supplemented media containing 10 ug/ml of iron saturated biotinylated holotransferrin (Sigma) for 1h to load the endosomal recycling compartment. Cells were chilled on ice and washed twice with cold PBS and once with low pH wash buffer (150 mM NaCl, 10 mM acetic acid pH 3.5) followed by two washes with cold PBS. Recycling was initiated by addition of complete medium supplemented with 1mg/ml of iron saturated holotransferrin (Sigma). After different time points, cells were lysed and processed for western blotting to quantitate the amount of transferrin in the endosomal recycling compartment. To quantitate recycled transferrin, a fraction of lysate and corresponding media was used to precipitate biotinylated transferrin using streptavidin agarose beads and processed for western blotting. Biotinylated transferrin was detected by incubating blots with streptavidin-HRP. Band intensities were quantitated by densitometry using Versa Doc (BioRad). Recycled transferrin at each time point was expressed as a percentage of total transferrin present in the cells and media.

### Endosome Recruitment Assay

Full length NDRG1 was PCR amplified using forward primer engineered with a T7 promoter and Kozak sequence for *in vitro* transcription and translation respectively. Amplified products were *in vitro* transcribed using T7 RNA polymerase and *in vitro* translated using reticulocyte lysate (Promega) in the presence of S^35^ methionine (GE). Recycling and early endosomes were isolated from HEK293 cells using step floatation sucrose gradient ultracentrifugation. Fractions at the 35%–25% interphase containing recycling and early endosomes were subjected to immunoisolation using Rab11 antibody (Zymed) bound to Miltenyi beads to purify recycling endosomes. Twenty five microliters of *in vitro* translated NDRG1 was incubated with Miltenyi beads bound recycling endosomes, 1 µg of GTPγS bound Rab4 and HEK293 cytosol (2 mg/ml) with appropriate controls (refer to Figure 3D) for 60min at 37°C. Endosomes were magnetically isolated, lysed using Laemmli buffer and the proteins were separated on a 4–15% SDS-PAGE gel and fluorographed.

### Lipid Overlay analysis

Lipid arrays (Echelon Biosciences) were blocked and probed with purified NDRG1flag (1 µg/ml) as per the manufacturer's instruction. The array was developed using ECL after probing with antibody against NDRG1.

### Live Cell Confocal Microscopy

HEK293 cells were transfected with NDRG1DsRed2 constructs using Lipofectamine 2000 reagent and stable clones were selected using G418. For recycling experiments HEK293-DsRed2 cells were plated on glass bottom petridishes (MatTek Corporation) allowed to adhere for 12 h. Cells were serum starved for 1h before being pulsed with Alexa flour-488 conjugated transferrin (45 µg/ml) for 5 min to load the early endosomes and 60 min to load the recyling endosomes. Recycling was initiated after washing cells in PBS and adding excess of unconjugated iron-saturated holo transferrin (1mg/ml). Live Cell confocal microscopy was carried out using Utraview LCI (Perkin Elmer) equipped with Spinning Nipkow disk with microlenses. Cells were viewed using a 100× objective. Images were grabbed every second in the temporal module, for 1–5 min using a LSI-cooled 12-bit CCD camera and processed using the NIH ImageJ software (http://rsb.info.nih.gov/ij/download.html) before being made into a movie. For experiments involving E-cadherin trafficking, E-cadherinEGFP constructs (a kind gift by Dr. James Nelson, Stanford University) were transfected in NDRG1DsRed2-HEK293 cells. Twenty four hours post transfection cells were imaged as above or chelated using EDTA (2.5 mM) for 30 min and plated on glass bottom petri dishes and then imaged as above.

## Supporting Information

Figure S1 NDRG1 interacts with recycling E-cadherin in DU-145 cells. Immunofluorescence analysis of DU-145 cells chelated with EDTA and replated on calcium-supplemented media immunoprobed for NDRG1 and E-cadherin after different time intervals. NDRG1 colocalizes with recycling E-cadherin. (Figure is a representative of three independent experiments.)
(7.29 MB TIF)

Figure S2 Purification of recombinant proteins. (A) Flag tagged NDRG1 produced in insect cells was purified by anion exchange chromatography, separated on SDS-PAGE gels and stained with Coomassie blue stain. Fraction of the purified proteins eluted out with 200mM and 300mM NaCl were analyzed by Western blotting (lower panel). (B) Rab4aGST produced in BL21 (DE3) cells was purified using glutathione agarose before being separated by SDS-PAGE and stained with Coomassie. Each lane shows successive elution of bound proteins using glutathione (15mM). The protein was confirmed by western blotting (lower panel).
(9.74 MB TIF)

Movie S1 Vesicular NDRG1 is located near the perinuclear region. HEK293 cells were transfected with pCMVNDRG1-DsRed2 vector. Stable cells were selected and imaged under Utraview LCI (Perkin Elmer). The movie show Z sections (0.15 µm) of cells from top to bottom. As seen, very few NDRG1 vesicles are located near the surface of cells and are mostly concentrated near the perinuclear region.
(6.89 MB AVI)

Movie S2 NDRG1 containing vesicles are motile and undergo fission and homotypic fusion. HEK293 cells were transfected with pCMVNDRG1-DsRed2 vector. Stable cells were selected and imaged under Utraview LCI (Perkin Elmer). Images were grabbed at the rate of 1image/sec and processed using ImageJ software before being made into a movie. NDRG1 vesicles in the perinuclear space also assume tubular morphology that rapidly moves from the perinuclear space towards the plasma membrane, a feature characteristic of recycling/sorting endosomal compartment.
(2.45 MB AVI)

Movie S3 Vesicular NDRG1 specifically interacts with recycling transferrin. NDRG1DsRed2-HEK293 cells were serum starved for 1h and incubated with Alexa flour-488 conjugated transferrin (45 µg/ml) for 60 min to load the recycling endosomes. Cells were then imaged under Utraview LCI (Perkin Elmer) live cell confocal microscope. Images were grabbed at the rate of 1image/sec and processed using ImageJ software before being made into a movie.
(0.35 MB MOV)

Movie S4 NDRG1 interacts with recycling E-cadherin. NDRG1DsRed2-HEK293 cells were transfected with pCMVE-cadherinEGFP construct. Twenty four hours post transfection calcium was chelated with EDTA (2.5mM) and cells were plated in calcium-supplemented media before being imaged. Cells were imaged using the Utraview LCI (Perkin Elmer) live cell confocal microscope. Images were grabbed at the rate of 1image/sec and processed using ImageJ software before being made into a movie. NDRG1 vesicles near the perinuclear space and close to the surface of the membrane localizes with E-cadherin as it is trafficked back to the cell surface.
(12.81 MB AVI)
