# Supplementary figures and images for: The N-Myc Down Regulated Gene1 (NDRG1) Is a Rab4a Effector Involved in Vesicular Recycling of E-Cadherin
Source: PLoS One. 2007 Sep 5;2(9):e844. doi: 10.1371/journal.pone.0000844 (PMC1952073; doi:10.1371/journal.pone.0000844)

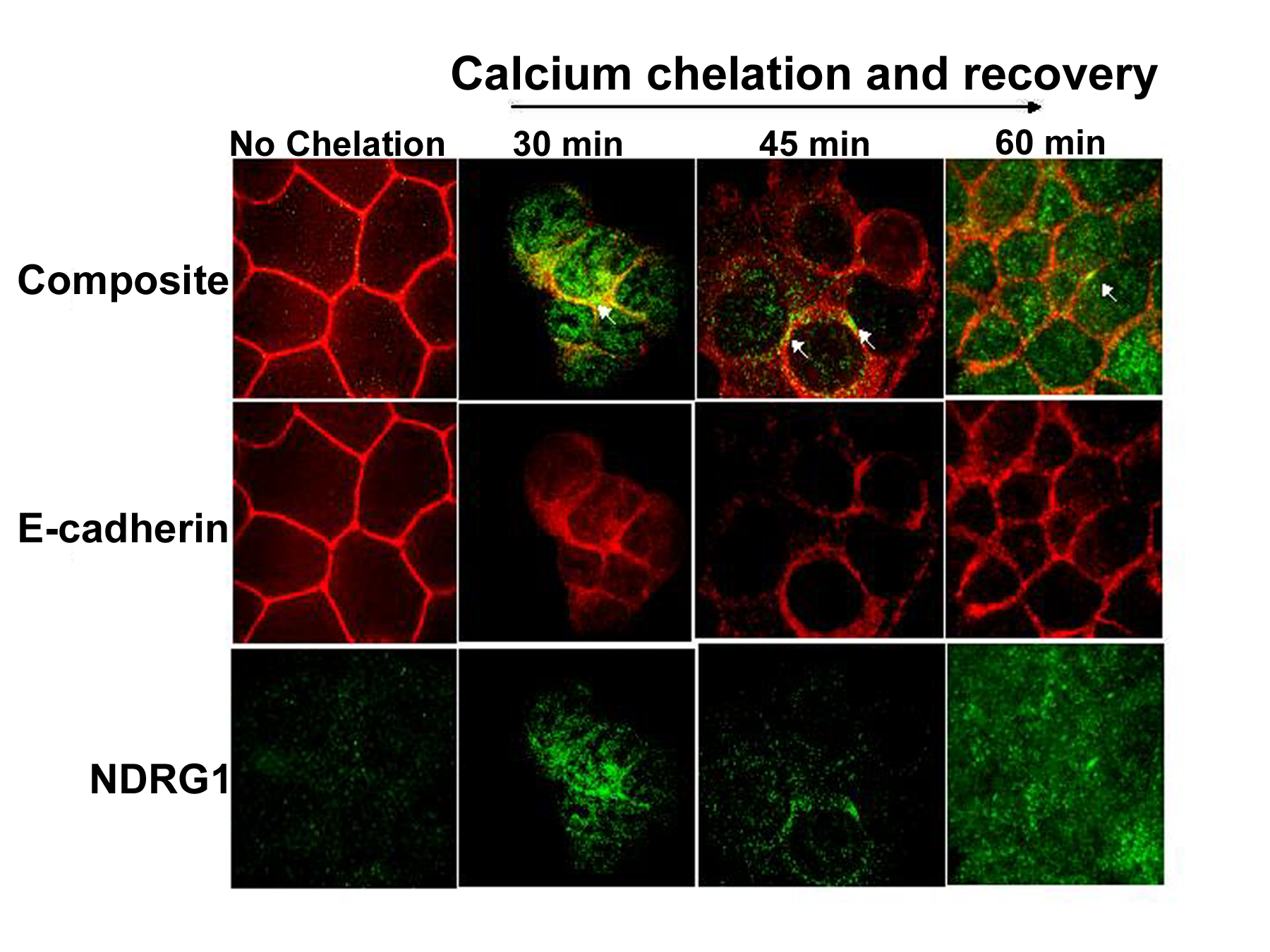

Supplement: Figure S1 — NDRG1 interacts with recycling E-cadherin in DU-145 cells. Immunofluorescence analysis of DU-145 cells chelated with EDTA and replated on calcium-supplemented media immunoprobed for NDRG1 and E-cadherin after different time intervals. NDRG1 colocalizes with recycling E-cadherin. (Figure is a representative of three independent experiments.) (7.29 MB TIF) [file pone.0000844.s001.tif]

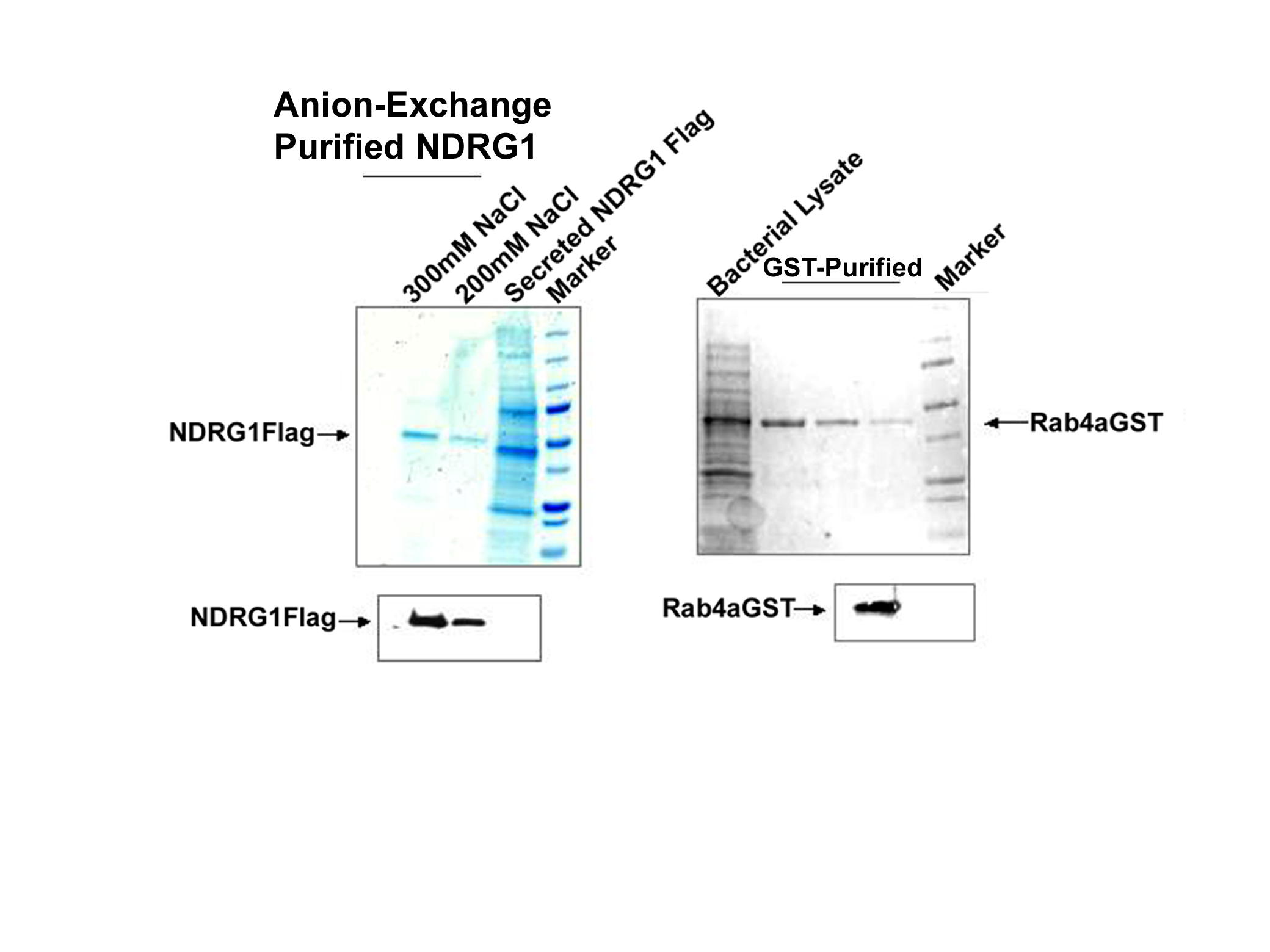

Supplement: Figure S2 — Purification of recombinant proteins. (A) Flag tagged NDRG1 produced in insect cells was purified by anion exchange chromatography, separated on SDS-PAGE gels and stained with Coomassie blue stain. Fraction of the purified proteins eluted out with 200mM and 300mM NaCl were analyzed by Western blotting (lower panel). (B) Rab4aGST produced in BL21 (DE3) cells was purified using glutathione agarose before being separated by SDS-PAGE and stained with Coomassie. Each lane shows successive elution of bound proteins using glutathione (15mM). The protein was confirmed by western blotting (lower panel). (9.74 MB TIF) [file pone.0000844.s002.tif]
